# Same Behaviors, Different Outcomes: Mothers’ and Fathers’ Observed Challenging Behaviors Measured Using a New Coding System Relate Differentially to Children’s Social-Emotional Development

**DOI:** 10.3390/children9050675

**Published:** 2022-05-06

**Authors:** Eric L. Olofson, Sarah J. Schoppe-Sullivan

**Affiliations:** 1 Psychology Department, Wabash College, Crawfordsville, IN 47933, USA; 2 Department of Psychology, The Ohio State University, Columbus, OH 43210, USA

**Keywords:** exploration, attachment, activation, socioemotional development, internalizing problems, externalizing problems, fathers

## Abstract

This study used a newly developed coding system for measuring the quality of parenting behavior to examine associations with children’s social-emotional development. The Risky Interaction Support and Challenge Scale (RISCS) measures the extent to which parents engage in behaviors that present physical and regulatory challenges to children, as well as parents’ tendency to allow children to pursue action goals autonomously. These behaviors were observed while parents (*n* = 57 fathers; *n* = 55 mothers; *n* = 50 pairs) interacted with their 1-year-olds who played on a structure that included a slide, a small climbing wall, and a tunnel. Trained raters reliably used the RISCS to measure several dimensions of parent behaviors related to children’s exploration, and all but one of the dimensions captured adequate variability in parent behavior. Although mothers and fathers did not differ in any of the dimensions, the associations between parent behavior and children’s social-emotional development did not overlap. Fathers who engaged in greater autonomy allowance and lower overprotection had toddlers with lower levels of internalizing behavior, whereas mothers who challenged children’s regulatory competence had toddlers with lower levels of externalizing behavior and greater competence. We discuss the implications of the findings for the literature on attachment theory and father-child relationships.

## 1. Introduction

From the beginning of Bowlby’s writings [1] on the nature and function of the attachment relationship, he emphasized the formative role of quality caregiving behavior in constructing secure attachments. His insights, tested and refined by Ainsworth and colleagues [2,3], elucidated how a caregiver’s sensitive response to a child’s distress provides that child with useful information about whom they can trust in times of stress. Research in this tradition has resulted in a rich and nuanced understanding of how this dynamic, reciprocal relationship forms [4], and the long-term outcomes associated with the quality of children’s trust in their caregiver as a secure base in times of stress [5].

However, as developmental researchers began to learn that the existing literature—built primarily on research about infants and their primary caregiver mothers [6]—did not explain father-child attachment relationships as well as mother-child attachment relationships [7], they began to call for a “wider view of attachment” [8] to better explain the form and function of father-child attachment relationships. These calls were motivated by theoretical [9,10] and empirical [11] work suggesting that fathers may play a more important role in children’s ability to take risks and explore, than they do in children’s desire to seek safe refuge in times of stress. In recent years, researchers have begun to retrace Ainsworth’s steps in identifying tasks that can elicit parent behaviors that promote children’s exploration. In this paper we introduce the Risky Interaction Support and Challenge Scale (RISCS) which measures parent behaviors that promote children’s desire and attempts to push the limits of their competence in risky exploration.

### 1.1. Traditional Research in Attachment Theory

Although Bowlby [1] and Ainsworth [12] emphasized the complementary functions of proximity-seeking and exploration, operationalizations of attachment-relevant parent behaviors have emphasized parent behaviors that build trust in the parent as a safe haven. Both the Strange Situation Procedure (SSP) and coding systems for the quality of attachment-related parent behaviors were tailored to measure the safe haven function of attachment relationships over the exploration function [8]. The most commonly used measurement system to assess the quality of parent behaviors [13,14] assesses parents’ skill in reading children’s behavioral and emotional cues, responding appropriately, avoiding adding to children’s distress by being intrusive, and setting a positive emotional tone. These behaviors signal to their children that they can be trusted in times of distress [15]. However, aside from one scale regarding the parent’s stimulation of the child’s development, these same behaviors are not as clearly important for children’s ability to confidently explore their surroundings and take the kinds of behavioral and intellectual risks that support cognitive and emotional development. Updated theories and operationalizations are needed to capture this aspect of parent-child relationships.

### 1.2. A Wider View of Attachment: Theory and Operationalizations

Calls to widen the view of attachment theory have emphasized the need to better measure quality support for children’s exploration behaviors. Given fathers’ greater tendency to engage in rough-and-tumble play with their children [16,17], one intriguing possibility is that fathers are more likely to focus their efforts on promoting exploration when the attachment system is not activated, than on providing a safe haven when it is. Therefore, the benefits of the wider view of attachment are twofold. First, research on the quality of parent support when children pursue challenging activities, engage in vigorous play, and take risks, may reveal unique developmentally beneficial effects on children. Second, by attending equally to parent behavior when children are distressed and seeking comfort and parent behavior when children are comfortable and ready to explore, developmental researchers can better understand the roles of mothers and fathers in fostering beneficial outcomes.

Two types of operationalizations of parent support for exploration have emerged. Groundbreaking research on the differential importance of SSP-measured attachment and support for exploration demonstrated the importance of assessing parenting behaviors that effectively support children’s secure exploration [11]. Grossmann and colleagues had mothers and fathers interact with their children in a cooperative, goal-directed play task and measured parent support for exploration using the Sensitive and Challenging Interactive Play (SCIP) Scale. The SCIP Scale was used to assign parents a single, global score that reflected their ability to present children with ability-appropriate challenges and support children’s attempts at autonomous solutions. Fathers’—but not mothers’—SCIP scores were unique and reliable predictors of later attachment security, providing initial evidence that support for exploration is an important part of attachment and, perhaps, a more valid assessment of father-child attachment than the SSP.

The validity of parental support for exploration is supported by findings that the quality of fathers’ support for exploration and risk-taking is predictive of children’s willingness to take age-appropriate risks [18]. More recent work by Majdandžić and colleagues [19] expanded coding of exploration support by introducing separate scales for parental overprotection, warmth, and challenging parenting behavior. This coding system assesses parental behaviors that support their children’s attempts at mastery, as well as parental behaviors that inhibit those attempts.

A second type of operationalization measured parent engagement in and support for play, an interaction context that is particularly important for father-child relationships [9,17,20,21]. Play—and especially rough-and-tumble play common among fathers in Western cultures [22]—introduces self-regulatory challenges for young children. Rough-and-tumble play arouses powerful emotions. In addition to intense pleasure, physical play can also elicit anger or sadness in a child if the play partner is too rough as well as frustration if the play partner tries to set limits on the child’s behavior. In these situations, children must learn to regulate their behavior and emotions in order to continue the largely pleasurable activity.

By measuring parent behaviors during play contexts, these operationalizations recognize that promoting secure exploration may play an important role in helping children develop mature self-regulatory strategies [8,9]. Fletcher, StGeorge, and Freeman [23] had father-child dyads play physical games while coders assigned fathers a global score on the Rough-and-Tumble Play Quality (RTP-Q) scale, which reflects a parent’s warmth, control during play, sensitivity, ability to balance winning and losing, and playfulness. Bureau and colleagues [24] used a relatively unstructured task—the Laughing Task, in which parents simply tried to make their children laugh—to elicit several behaviors related to the wider view of attachment: physical proximity, appropriate parental effort, following the child’s rhythm (the opposite of intrusiveness), and focus on the dyadic interaction.

The key advancement of both types of operationalizations of parental support for exploration is that they posit a role for parents during exploration. In contrast to Bowlby’s approach that saw children using the parent as a secure base for exploration, current approaches emphasize the parent’s ability to encourage children to push their behavioral and regulatory competencies further than children could do on their own.

### 1.3. Exploration Support and Child Outcomes

A burgeoning literature demonstrates that parental support for exploration can predict positive child outcomes [25]. Fathers’ scores on both the SCIP scale and the Laughing Task have been associated with children’s attachment representations, a set of findings consistent with the theoretical argument that exploration support is more central to father-child attachment than sensitive responsiveness to distress.

Beyond the relationship with attachment, the wider view of attachment has received additional support from findings of associations between fathers’ exploration support and children’s emotional development. Children who are supported in exploration learn to trust in their ability to overcome challenges rather than respond to roadblocks by becoming anxious [26]. Parental—especially paternal—challenging behavior predicts low levels of child anxiety [27,28,29]. The converse may also be true; parents who are overprotective have more anxious children than parents who are low in overprotective behavior [30].

This association between parental challenge and children’s internalizing problems also holds when researchers have examined children’s willingness to take developmentally appropriate risks. Children who are “activated” [9] to take physical risks in their father’s presence have fewer internalizing problems than children who are either risk-averse or reckless [31,32]. The view that fathers’ rough-and-tumble play is a rich context for activating children’s desire to take physical risks is supported by the increasing number of studies finding that fathers’ rough-and-tumble play is associated with positive outcomes in children [33]. High quality parental engagement in rough-and-tumble play predicts fewer behavioral [23] and emotional [34] problems.

### 1.4. Limitations of Existing Coding Systems

Despite the growth in systems for coding parent support for exploration, two limitations in the existing literature motivated the current study. First, existing coding systems generally reserve high scores for behaviors that are sensitive (but see [19] for an exception). However, it is still an open question whether parent behaviors central to the secure base function of attachment relationships are also central to the exploration support function. For example, challenging children to push beyond their current abilities to acquire more advanced skills may necessarily be intrusive, a behavior that is incompatible with sensitive caregiving in traditional coding systems [14]. Similarly, when children are making progress toward a challenging goal on their own, it may be beneficial for parents to avoid interacting with their children so that they can diagnose and solve problems on their own and practice regulating any frustration that arises during this process. This potentially positive parental behavior would be coded as detachment—and thus a lack of sensitivity—in traditional coding systems. In some systems for measuring exploration support, parents’ active support for children’s autonomy is coded [19,35] but none include unique codes for parents’ willingness to adopt a stance of nonintervention. For example, Majdandžić and colleagues [19,35] coding system includes a scale that separates behavior that either actively encourages autonomy or takes it away through intrusive behavior. In their coding system, simply adopting a stance of watchful nonintervention is considered a mild form of challenging parenting behavior.

The second limitation in the current literature is that in existing systems for measuring parent support for exploration, either parents’ attempts to challenge children’s behavioral skill or to activate their regulatory systems through play are coded. No existing coding systems have separate scales to measure parents’ ability to challenge their children’s behavioral competence and their regulatory competence. For example, the challenging parenting behavior scale in Majdandžić and colleagues’ [19] system captures both rough-and-tumble-play and encouragement to perform more difficult tasks. These two different types of behavior are both challenging but are conceptually distinct. Rough-and-tumble play destabilizes children, thus challenging their ability to maintain emotional and behavioral self-regulation [9]. In contrast, challenging children to perform difficult tasks stimulates their cognitive development and scaffolds their behavioral competence. In light of the lack of a coding scheme that distinguishes these types of challenges, it is not clear whether these conceptually distinct types of parental challenge are differentially associated with child outcomes.

### 1.5. The Current Study

The purpose of the current study was to test the reliability and validity of the newly-developed Risky Interaction Support and Challenge Scale (RISCS) [36]. The RISCS was influenced by the system developed by Majdandžić and colleagues [19]. We incorporated the Overprotection scale from their system and used their Challenging Parenting Behavior scale as the basis for the Challenging Behavioral Competence scale in the RISCS, including the definition of those constructs (see Appendix A). Due to the emerging findings that fathers’ exploration support may impart developmental benefits to children, the RISCS separated parents’ ability to challenge children’s regulatory competence, out of Challenging Parenting Behavior into a new scale called Challenging Regulatory Competence. We also introduced a second scale called Autonomy Allowance for coding parents’ adoption of a stance of nonintervention to allow the child to act autonomously.

In contrast to traditional parent coding systems [13,14] and certain exploration support scales [11], the RISCS does not reserve high scores for behaviors that are clearly sensitive. Behavior that may lead to high intrusiveness and low sensitivity scores in traditional systems, but which successfully challenges the child’s behavioral or regulatory competence, may earn high scores for those dimensions in the RISCS. Likewise, behavior that may lead to high detachment and low sensitivity scores in traditional systems, may earn high scores for that dimension in the RISCS if it allows the child to act autonomously.

We tested the RISCS on parent interactions with their one-year-old children while those children were playing on a toy that invites mild physical risks. Mothers and fathers were observed playing with their children in a room containing a climber toy. The climber toy presented mild physical risks to children as they climbed steps on one side and used a slide on the other end. This is a popular toy and thus presents an ecologically valid context in which to observe parent-child interactions that involve more physical risk than is typical in studies that investigate sensitive parent behavior.

The current study was motivated by five research questions: (1) Will coders achieve adequate interrater reliability using the RISCS when coding both fathers and mothers? (2) Does the RISCS appear to capture variability in behaviors engaged in by fathers and mothers during the climber task? (3) What similarities and differences exist between mothers and fathers in behaviors coded by the RISCS? (4) Are children’s characteristics (i.e., gender and temperament) associated with fathers’ and mothers’ behaviors coded using the RISCS? (5) Are RISCS scores of mothers and fathers related to children’s social-emotional development, and do these associations differ for mothers and fathers?

## 2. Materials and Methods

### 2.1. Participants and Procedure

Data were drawn from a longitudinal study of child and family development in dual-earner families in a large city and surrounding area in the Midwestern United States. Different-sex couples expecting their first biological child were recruited during the third trimester of pregnancy from childbirth education classes and via advertisements in doctors’ offices and newspapers, and through snowball sampling and word-of-mouth. To be eligible for participation, expectant parents had to be at least 18 years old, married or cohabiting, working full time and planning to return to work postpartum, and able to read and speak English. As compensation for participating at each wave of the study, participants received small incentives in the form of cash, gift cards, and infant books or toys.

The original sample consisted of 182 couples. The data used in this report come from a longitudinal follow-up that focused on a subsample of toddlers (*n* = 62) and their parents (*n* = 112 parents; 57 fathers; 55 mothers; 50 matched mother-father pairs) who participated in two laboratory assessments spaced one month apart when the child was approximately 12–18 months old. Which parent visited the lab first with their toddler was counterbalanced. As part of these laboratory assessments, each parent and child participated in a 5-min video recorded episode in which the parent and child were introduced to a play structure that included a slide, small climbing wall, and a tunnel. The parent was asked to encourage their child to try the different things they were able to do on the play structure. At the mother-child assessment, mothers also completed the ITSEA [37], a survey measure of toddler social-emotional development, described below.

The *n* = 62 participating toddlers were age 16.37 months on average (*SD* = 1.39), comprising 40 boys and 22 girls. At recruitment, children’s mothers were 27.90 years old on average (*SD* = 4.11), and 89% identified as White, 5% as Black or African American, 3% as mixed race, and <2% each identified as Asian or another race. Less than 2% of mothers identified as Hispanic. At recruitment, children’s fathers were 29.40 years old on average (*SD* = 3.94), and 87% identified as White, 5% as Black or African American, 3% as Asian, and <2% each identified as Pacific Islander, mixed race, or another race. Three percent of fathers identified as Hispanic. Overall, 81% of mothers and 73% of fathers had a bachelor’s degree or higher-level education. Median annual family income at recruitment was $79,500 and 87% of couples were married. Demographic characteristics of the parents and children who participated in the toddlerhood follow-up were similar to those in the larger sample. There were no significant differences between parents who participated and those who did not in terms of marital status, family income, race/ethnicity, age, or education. The only significant difference was for child gender (chi-square = 7.34, *df* = 1, *p* = 0.007), such that participating children in the toddler follow-up were more likely to be boys compared with children who did not participate in the toddler follow-up. The larger number of boys than girls at the toddler follow-up was not explained by other demographic variables. However, comparisons between families of boys and girls in the original sample on involvement in childcare from 3 to 9 months postpartum found that fathers of boys were more involved in caring for their infants than fathers of girls (further details available from the authors upon request). It is thus possible that fathers of boys were more motivated to continue participating in the study.

### 2.2. Measures

#### 2.2.1. Risky Interaction Support and Challenge Scale (RISCS) Coding

The RISCS uses a series of 5-point ratings to capture aspects of parent behavior relevant to supporting children’s developmentally appropriate increasing desire for independent exploration and achievement. The 5 min observed climber task episodes with mothers and fathers were coded for the quality of parents’ parenting behaviors by trained raters. The complete RISCS is provided in Appendix A [36]. In brief, the parenting behaviors coded include *challenging behavioral competence* (physical, expressive), which reflects the extent to which the parent encourages the child to go outside their comfort zone to expand their skills and achieve their goals; *challenging regulatory competence*, which captures parents’ efforts to challenge children’s ongoing self-regulation or encourage the child’s regulatory efforts; *overprotection* (expressive, physical), which reflects the extent to which the parent conveys exaggerated worry or concern for the child’s wellbeing and safety in the absence of legitimate risk; and *autonomy allowance*, or parent behavior that permits children to pursue activities that are outside of their comfort zone, beyond their current abilities, or contravene typical expectations of behavior, by simply attending to the child’s activities while adopting a stance of non-intervention.

The authors, the developers of the RISCS, trained three coders to rate each parent-toddler interaction according to each of these parent behaviors. Coders were unaware of the hypotheses concerning associations with child characteristics. They first practiced identifying codable behaviors on videotaped parent-child interactions from a different study. Next, coders established reliability using the RISCS on a set of six videos of parent-child interactions (three with mothers, three with fathers) from the current study that had already been coded by the authors with perfect agreement. After an initial round of coding, the first author and the coders discussed which behaviors were seen as codable in the current study but did not discuss scores. Coders then re-coded the six pilot videos and repeated the process until all scores were within one point of the authors’ scores and intraclass correlation coefficients were above 0.80. After achieving this level of reliability, the rest of the videos were double-coded. When scores differed by one point, the average rating was used. When scores differed by more than one point, discrepancies were resolved in discussion with one of the authors. Interrater reliabilities across the entire sample are reported in the Results section.

#### 2.2.2. Infant-Toddler Social-Emotional Assessment

Mothers completed the Infant-Toddler Social-Emotional Assessment (ITSEA) [37,38], a reliable and valid assessment tool appropriate for children aged 12–48 months and designed to identify competencies and areas of concern in toddlers’ social–emotional development across four broad domains: Competence, Internalizing, Externalizing, and Dysregulation. All items were rated on a scale of 0 to 2, where 0 = Not true/rarely, 1 = Somewhat true/sometimes, and 2 = Very true/often. Competence (37 items; α = 0.85) includes aspects such as compliance, attention regulation, imitation and pretend play skills, mastery motivation, empathy, emotional awareness, and prosocial peer behaviors. Internalizing (32 items, α = 0.73) reflects depression, social withdrawal, anxiety, separation distress, and extreme inhibition/shyness, whereas Externalizing (24 items, α = 0.79) reflects high activity, impulsivity, aggression, and defiance. Dysregulation (34 items, α = 0.81) captures problems in sleeping and eating, problems regulating negative emotional states with respect to reactivity and regulation, and unusual sensory sensitivities.

#### 2.2.3. Infant Temperament

At 3-months postpartum, mothers reported on children’s surgency (13 items; α = 0.83), negative affect (12 items; α = 0.77), and effortful control (12 items; α = 0.65) via the Revised Infant Behavior Questionnaire–Very Short Form [39]. Each of the 37 items required mothers to rate on a scale of 1 to 7 the extent to which children exhibited a particular behavior, where 1 meant that the parent never observed their infant exhibiting the behavior and 7 meant the behavior was very frequently observed. Mothers could also select “NA” if they had not observed their infant in the situation described during the last week. Item responses were averaged to create scores for each dimension of temperament.

## 3. Results

### 3.1. Analysis Plan

First, coders’ reliability in applying the RISCS scales to the observed father- and mother-toddler interactions was assessed using percent agreement within one point and intraclass correlations. Second, means, standard deviations, and ranges of the RISCS scales were inspected to describe the distributions of parents’ behaviors in this sample. Third, correlations, paired-samples *t*-tests, and chi-square tests were used to assess similarities and differences in father and mother behaviors captured by the RISCS. Fourth, associations of children’s characteristics (temperament and gender) with mothers’ and fathers’ RISCS scores were computed. Finally, correlations between fathers’ and mothers’ RISCS scores were calculated to examine relations between parents’ behaviors and children’s social-emotional adjustment, Fisher’s *r*-to-*z* tests were used to compare corresponding correlations for fathers and mothers, and these correlations were recomputed controlling for mothers’ reports of infant temperament at 3 months postpartum.

### 3.2. Reliability and Distribution of RISCS Scores

Interrater reliability is reported in Table 1. Percent agreement within one scale point ranged from 81–100% and was similar for fathers’ and mothers’ behaviors. With the exception of the expressive overprotection scale, coders achieved strong intraclass correlations, ranging from 0.791 to 0.900, which was similar in strength for fathers and mothers. Moreover, the descriptive RISCS statistics (except expressive overprotection) reflected the fact that these scales appeared to capture adequate variability in parent behavior. Reliability was low for expressive overprotection because of its restricted range; moderate to high levels of this behavior were observed for neither fathers nor mothers.

### 3.3. Similarities and Differences between Fathers and Mothers

Correlations between corresponding RISCS scores for fathers and mothers (Table 2) revealed one significant association: fathers’ scores on autonomy allowance were positively associated with mothers’ scores on autonomy allowance, *r* = 0.378, *p* < 0.01. The other corresponding correlations ranged from −0.095 to 0.174 and did not reach statistical significance. Notably, for both fathers and mothers, higher scores on overprotection were related to lower scores on autonomy allowance, and higher scores on challenging behavioral competence were also related to lower scores on autonomy allowance.

Paired *t*-test analysis revealed no statistically significant differences in the mean values for fathers’ and mothers’ RISCS behaviors (Table 1). However, follow-up analysis further considered the distributions of RISCS scores for fathers and mothers (with the exception of expressive overprotection, which had inadequate variability), and used chi-square tests to examine whether very high scores were more characteristic of one parent or the other. On each of the other four scales (challenging behavioral competence, challenging regulatory competence, physical overprotection, and autonomy allowance), fathers and mothers were divided into groups on the basis of whether they received high scores (4 s or 5 s) or lower scores. Of the four scales examined, there was a significant difference in the distribution of fathers’ and mothers’ scores on challenging regulatory competence, χ^2^(1) = 3.99, *p* = 0.046. Fathers were more likely to receive high scores on challenging regulatory competence (*n* = 10 of 57) than were mothers (*n* = 3 of 55).

### 3.4. Children’s Characteristics and RISCS Scores

Prior to examining relations between children’s characteristics and RISCS scores, the physical and expressive overprotection scales were summed (separately for fathers and mothers) in order to provide an overall score for overprotection with adequate variability. Independent sample *t*-tests considered whether fathers’ and mothers’ RISCS scores differed for boys versus girls. No statistically significant differences were observed, with *p*-values ranging from 0.167 to 0.970. Correlations of fathers’ and mothers’ perceptions of infant temperament at 3 months postpartum (i.e., surgency, negative affect, and effortful control) with fathers’ and mothers’ RISCS behaviors also revealed no statistically significant associations. For fathers and mothers, these correlations ranged in absolute value from 0.01 to 0.19.

### 3.5. Relations between RISCS Scores and Toddlers’ Social-Emotional Adjustment

Correlations between fathers’ and mothers’ RISCS scores and toddlers’ social-emotional adjustment are shown in Table 3. Fathers who engaged in greater autonomy allowance had toddlers with lower levels of internalizing behavior, *r* = −0.28, *p* < 0.05. In contrast, fathers who showed higher combined physical and expressive overprotection had toddlers with higher levels of internalizing behavior, *r* = 0.34, *p* < 0.01. When mothers were observed to challenge children’s regulatory competence more strongly, their toddlers demonstrated lower levels of externalizing behavior, *r* = −0.32, *p* < 0.05, and greater competence, *r* = 0.29, *p* < 0.05.

For the *n* = 50 subsample of families in which we had parent behavior data from matched pairs of mothers and fathers and ITSEA data on toddlers, we were able to further follow up and test whether the strength of the pairs of associations were significantly different using Fisher’s *r*-to-*z* test for comparison of correlations from dependent samples. The associations of challenging regulatory competence with children’s competence were significantly different for mothers (*r* = 0.30) and fathers (*r* = −0.14), *z* = 2.42, *p* = 0.008; however, the associations for challenging regulatory competence and children’s externalizing were not (*r*_m_ = −0.31, *r*_f_ = −0.17, *z* = −0.78, *p* = 0.216). The associations of autonomy allowance and children’s internalizing were not significantly different for fathers (*r* = −0.30) and mothers (*r* = −0.17), *z* = −0.84, *p* = 0.201, but the associations of overprotection with children’s internalizing were significantly different for fathers (*r* = 0.39) and mothers (*r* = 0.08), *z* = 1.68, *p* = 0.047.

Finally, in light of anticipated and significant associations between mothers’ perceptions of infant temperament at 3 months and toddlers’ social-emotional adjustment (Table 4), we re-ran the correlations between parents’ RISCS scores and toddlers’ ITSEA scores controlling for mothers’ reports of children’s surgency, negative affect, and effortful control at 3 months postpartum. These partial correlations revealed that three of the four significant associations between parents’ RISCS scores and toddlers’ social-emotional adjustment retained their statistical significance even when controlling for mothers’ reports of infant temperament. The exception was the correlation between mothers’ challenging regulatory competence and toddlers’ externalizing behavior, which dropped below *p* < 0.05 when controlling for mothers’ reports of infant temperament, *pr* = −0.27, *p* = 0.064.

## 4. Discussion

The current study investigated the reliability and validity of a newly developed coding system for measuring parents’ support for exploration with their young children. We found that coders could rate reliably the behaviors captured in the RISCS, including autonomy allowance, which focuses on the parent’s lack of interference in the child’s activities. In addition, we found that fathers’ and mothers’ scores on the RISCS were largely similar. We also found that parents’ RISCS scores were associated with children’s social and emotional development. Consistent with our predictions and with previous research e.g., [29], fathers’ lower levels of overprotection and higher levels of autonomy allowance were associated with lower levels of internalizing problems in children. Finally, we found unexpected associations between higher levels of maternal challenging regulatory competence and lower externalizing problems and higher competence in toddlers. Taken together, these patterns suggest that the RISCS captures exploration-relevant parenting behaviors that are similar between parents but have different associations with child outcomes.

These data contribute to the burgeoning scholarship on parental support for children’s exploration and on father-child relationship quality. One important theoretical advance lies in the differential conception of what it means when parents refrain from involving themselves in children’s ongoing activities. Coding scales of parent behavior from the attachment tradition treat such instances as evidence of parental detachment, or being “emotionally uninvolved or disengaged and unaware of the child’s needs for appropriate interaction” [14]. Detachment in the context of the safe-haven function of attachment is associated with poorer child outcomes [40], but the current findings suggest that allowing autonomy by “attending to the child’s activities while adopting a stance of non-intervention” may be an important protective factor for children by supporting healthy risk-taking in the context of the exploration function of attachment. Similarly, the positive relation between paternal overprotection and children’s internalizing problems is consistent with other studies [41] and with the view that overprotection is a risk factor in children’s development. When it comes to children’s autonomous exploration activities, it may be best for fathers to err on the side of non-intervention.

The current study is broadly consistent with the empirical literature in finding that when fathers demonstrate high-quality parenting behaviors, their children are less likely to have internalizing problems. Low paternal overprotection and high autonomy allowance were associated with fewer internalizing problems in children. This pattern fits with empirically-based models of the etiology of anxiety that emphasize the father’s role in opening children to the world and promoting their independence [42]. Notably, no other variables in the current study explained a significant amount of variance in internalizing problems, although interpretations regarding the uniqueness of fathers’ roles must be tentative because differences in statistical significance do not entail differences in relations between constructs [43].

Although the findings regarding fathers’ behavior and children’s internalizing problems are broadly consistent with the empirical literature, there was one clear difference. Other studies have found that fathers’ challenging parenting behavior is associated with fewer anxiety symptoms in their children [28,44], a finding that did not emerge in the current study. One plausible explanation lies in the different operationalizations of children’s behavior problems. Previous studies investigating challenging parenting behavior have focused on child anxiety, whereas the current study used a scale for internalizing problems that included depression, extreme shyness, and social withdrawal. A second plausible explanation for the differences lies in the age at which internalizing problems were measured. Children in the current study were tested between 12-and 18-months, whereas previous studies have focused on children’s anxiety later in later preschool years. Not only might mothers of toddlers have struggled to report anxiety symptoms as distinct from other related behaviors, but also theoretical models of the relation between fathers’ parenting and child anxiety emphasize the importance of those effects as children mature and gain independence [8,9,42]. It is possible that anxiety-specific effects do not emerge until the later preschool years.

The lack of association between paternal scores on challenging regulatory competence (CRC) and child outcomes was surprising, because the RISCS places physical play within the CRC subscale. Empirical [23,28,31,33] and theoretical [8,9,42] studies have found that quality rough-and-tumble play between fathers and children is associated with positive outcomes in children. There are several possible explanations for why these relations did not emerge in the current study. First, it is possible that that paternal CRC at 16 months does not serve the exploration function of attachment, and that these relations emerge later in the child’s life. Second, the perceptual salience of the climber toy in the room may have dictated the nature of the play and made it difficult for fathers to engage in more open-ended physical play. Third, it is possible that parental encouragement of children’s own regulatory efforts, which comprises part of the CRC subscale, is not related to paternal activation of risk-taking or rough-and-tumble play. This may have resulted in some fathers who do not typically engage in physical play with children scoring highly on CRC.

Comparisons between mothers’ and fathers’ results are noteworthy for several reasons. Mothers and fathers scored similarly on each of the RISCS subscales, a finding that is consistent with other comparisons between mothers’ and fathers’ exploration-relevant behaviors with first-born children [24,29]. However, none of the significant correlations between RISCS subscales and child outcomes overlapped between mothers and fathers, raising the possibility that the same parenting behaviors in mothers and fathers may have different behavioral consequences for children. Although this explanation must be treated with caution, as differences in significance do not entail significant differences, Fisher’s *r*-to-*z* tests found that two of these pairs of correlations differed significantly between parents. First, fathers’ overprotection, but not mothers’, was associated with children’s internalizing problems. This pattern would make sense if fathers in the current study were more likely than mothers to encourage their children’s risk-taking and exploration; overprotection in that role is likely to be more detrimental to children than overprotection by the parent serving as the child’s safe haven in times of distress [9,31].

The other significant difference in RISCS-to-outcomes correlations was that mothers’ CRC, but not fathers’, was associated with greater competence in children. This finding was unexpected and is more difficult to explain using the existing literature on father-child interaction. One possibility is that high and low scores on CRC reflect different kinds of behaviors. Lower-to-moderate levels of CRC may reflect variability in parental engagement and stimulation of development. If this is true, then the relation between mothers’ CRC and children’s competence may have been driven by variability in maternal engagement. As nearly all of the mothers scored within this lower range of CRC, there was a sufficient sample size to uncover relations with child competence. In contrast, perhaps only higher scores reflect behaviors that are sufficiently challenging to children’s regulatory systems. Consistent with theories positing that fathers often fill this role [9], post-hoc analyses confirmed that fathers were significantly more likely than mothers to score highly on CRC. However, it is possible that not enough fathers scored in this range to test associations with child outcomes. Therefore, it is possible that the intensity of challenges changes their developmental significance, with gentle challenges relating to sensitive engagement within the safe-haven context of attachment, and more intense regulatory challenges relating to exploration and risk-taking.

The current study had several limitations. The demographics of the study sample limit the generalizability of the findings. Families in the current study were all heterosexual parents raising their first child in a dual-earner, cohabiting household. Preliminary research with homosexual fathers suggests that in those households, like those led by heterosexual parents, primary caregivers act as safe havens and secondary caregivers act to support exploration [45]. These data suggest that the patterns in the current study may apply to primary and secondary caregiving gay male fathers, but this is speculative. Regarding the child’s status as first-born, it is possible that parents’ exploration-supportive behavior may be different with later-born children [29]. The limited range of socioeconomic status and ethnicity limit the study’s generalizability to lower-income and BIPOC samples. For example, fathers with more education spend more time interacting with children [46], which may have contributed to the lack of parent-gender differences in RISCS scores. However, the limited research on parental support for exploration using samples from a broad range of socioeconomic status makes it difficult to hypothesize precisely what patterns might be expected [25]. It is also possible that the overrepresentation of boys in the sample meant we had especially involved fathers participating, which could further limit the generalizability of the findings. The sample size was modest, which limited the feasibility of factor analytic and other multivariate analyses. Despite that limitation, given the inclusion of much-needed observational data on fathers’ behavior [6], and the need for development and validation of additional measures of parental support for exploration, the findings are noteworthy. It is important to state that the current study relied on uncorrected zero-order correlations to answer the research questions. This decision was made because the purpose of the current study was not to test theories, but rather to introduce a novel tool for researchers and to limit Type II errors when suggesting avenues for additional research. Therefore, there is a risk that some of the findings reflect Type I errors. Finally, child outcomes were measured concurrently with parent behavior, so no firm claims regarding the direction of relations can be made, although controlling for infant temperament does strengthen the claim that parent behavior in support of exploration contributes to children’s social-emotional development.

Findings from the current study suggest several directions for future research. Given the theoretical importance of exploration support in the preschool years and beyond, future studies using the RISCS should examine behavior in older children engaging in riskier activities. This would give overprotective more opportunities for parents to display those behaviors, and opportunity to investigate their relations with child outcomes. Including older children would also help address the appropriate way to assess overprotection. In the current study and in other studies using observational measures of overprotection e.g., [19], expressive and physical overprotection were combined. Future research should investigate whether the method of measuring overprotection is theoretically meaningful or if it is simply a byproduct of other factors such as context and child age. It is also important to recruit a more diverse sample. The sample used in the current study was originally recruited specifically to investigate the transition to parenthood in dual-earner couples, so future studies investigating parental support for risk-taking specifically should take care to broaden the demographic characteristics of the sample. Although coders in the current study achieved strong reliability when coding videotaped parent behavior with toddlers, it is unclear whether the RISCS could be used reliably to code more intense expressive overprotection, live behavior, or parent behavior during interactions with older children. Longitudinal studies and studies with larger sample sizes will help assess the direction of relations between parent behavior and child outcomes, and will enable more robust model-testing approaches. Factor analyses will be especially important to address whether challenging children’s behavioral and regulatory competencies should be considered as one or two constructs.

## 5. Conclusions

The current study adds to the literature on parental support for children’s exploration-relevant behaviors and the associations between those behaviors and child outcomes. The RISCS appears to be a reliable and valid measure of parenting behavior for fathers and mothers. The study contributes to research in this tradition in three distinct ways. First, the findings suggest that in the context of children’s exploration, simply attending to children’s ongoing activity while taking a stance of non-intervention may support children’s development. Second, the findings extend the literature on the connection between paternal exploration support and children’s internalizing problems, by including toddlers in the study results. Third, the findings provide a nuanced picture of similarities and differences between mothers and fathers, and thus challenge the idea that mothers’ and fathers’ roles are necessarily linked with gender.

## Figures and Tables

**Table 1:** Interrater reliability for RISCS, descriptive statistics, and mother–father comparisons.

	Percent Agreement within 1 Point		Intraclass Correlation Coefficients		Means (*SD*) ^2^		Ranges		Paired *t*-Value	*p*-Value
RISCS Subscale	Fathers	Mothers	Fathers	Mothers	Fathers	Mothers	Fathers	Mothers		
PCBC ^1^	87.8	95.2	0.791	0.876	1.94 (0.93)	1.98 (0.84)	1.00–4.00	1.00–4.00	−0.60	0.550
CRC	95.1	95.2	0.857	0.900	2.18 (1.33)	1.80 (0.93)	1.00–5.00	1.00–4.50	1.65	0.104
PO	90.2	95.2	0.796	0.873	1.54 (0.91)	1.77 (1.05)	1.00–5.00	1.00–5.00	−1.23	0.222
EO	100	97.6	0.500	N/A	1.12 (0.26)	1.11 (0.23)	1.00–2.50	1.00–2.00	−0.70	0.489
AA	82.9	81.0	0.852	0.845	3.29 (1.12)	3.16 (1.15)	1.00–5.00	1.00–5.00	0.82	0.415

^1^ PCBC = Physical Challenging Behavioral Competence; CRC = Challenging Regulatory Competence; PO = Physical Overprotection; EO = Expressive Overprotection; AA = Autonomy Allowance. ^2^ *N* = 57 for fathers and *N* = 55 for mothers. *N* = 50 and *df* = 49 for paired comparisons.

**Table 2:** Intercorrelations among RISCS scores.

RISCS	1	2	3	4	5	6	7	8
Fathers								
1. PCBC ^1^	--							
2. CRC	0.05	--						
3. OP	−0.04	−0.22	--					
4. AA	−0.26 *	0.18	−0.59 ***	--				
Mothers								
5. PCBC	0.06	−0.16	0.24	−0.10	--			
6. CRC	0.03	0.17	0.04	−0.01	0.09	--		
7. OP	0.25	−0.28 *	0.12	−0.31 *	0.11	−0.27 *	--	
8. AA	−0.31 *	0.29 *	−0.24	0.38 **	−0.31 *	0.06	−0.70 ***	--

^1^ PCBC = Physical Challenging Behavioral Competence; CRC = Challenging Regulatory Competence; OP = Overprotection (physical and expressive combined); AA = Autonomy Allowance. * p < 0.05. ** *p* < 0.01. *** *p* < 0.001. *N*s range from 50 to 57. Expressive and Physical Overprotection scores were combined due to low variability in Expressive Overprotection.

**Table 3:** Correlations between RISCS scores and toddler social-emotional development.

	ITSEA Domains			
RISCS Subscale	Externalizing	Dysregulation	Internalizing	Competence
Fathers				
PCBC ^1^	−0.04	−0.03	0.10	0.06
CRC	−0.18	−0.10	−0.07	−0.16
OP	0.01	0.12	0.34 **	0.01
AA	0.00	−0.13	−0.28 *	−0.12
Mothers				
PCBC	−0.02	−0.07	0.23	0.12
CRC	−0.32 *	0.00	0.14	0.29 *
OP	−0.03	−0.02	0.05	0.02
AA	0.13	0.03	−0.15	−0.21

^1^ PCBC = Physical Challenging Behavioral Competence; CRC = Challenging Regulatory Competence; OP = Overprotection (physical and expressive combined); AA = Autonomy Allowance. * *p* < 0.05. ** *p* < 0.01. *N*s range from 55 to 57. Expressive and Physical Overprotection scores were combined due to low variability in Expressive Overprotection.

**Table 4:** Descriptive Statistics and Intercorrelations of Infant Temperament and ITSEA scores.

	1	2	3	4	5	6	7	Means (*SD*)
Infant Temperament								
1. Surgency	--							3.81 (0.85)
2. Negative Affect	0.07	--						3.42 (0.86)
3. Effortful Control	0.38 **	−0.17	--					5.44 (0.54)
ITSEA Scores								
4. Externalizing	0.14	0.31 *	−0.09	--				0.48 (0.23)
5. Dysregulation	0.00	0.41 ***	−0.05	0.45 ***	--			0.38 (0.20)
6. Internalizing	0.11	0.18	−0.24	0.07	0.25 *	--		0.52 (0.16)
7. Competence	0.22	−0.27 *	0.32 *	−0.09	−0.09	−0.04	--	1.31 (0.23)

Note: * *p* < 0.05. ** *p* < 0.01. *** *p* < 0.001. *N* = 62.

## Data Availability

The data presented in this study are available on request from S.J.S.-S. The data are not publicly available due to the need to protect confidentiality of participants.

## Appendix A

The Risky Interaction Support and Challenge Scale:

The Risky Interaction Support and Challenge Scale (RISCS) is designed to allow coding of parent behavior during periods in which children are engaged in tasks that involve physical risk and/or behavioral challenge. The four scales capture aspects of parent behavior relevant to supporting children’s increasing desire for independent exploration and achievement.

These scales are meant to accompany the Qualitative Ratings of Parent-Child Interaction (colloquially “NICHD Scales”) developed by the NICHD [13] and most recently by Cox and Mills-Koonce [14], although the RISCS may be used independently. The rating procedures are similar to those used in the NICHD Scales. After coders are familiar with the breadth of behaviors in a given task, they should (1) watch a tape once while taking minimal notes; (2) watch the tape a second time while taking careful longhand notes that identify codable behaviors, the time stamp at which the behaviors occurred, and the intensity of each behavior; (3) assign an initial score for each dimension; (4) watch the tape a third time to consider the initial scores; (5) assign a final score for each dimension; and (6) watch the tape a fourth time to consider the scores. Note that for both the challenging competence and overprotection scales, only observed behaviors are coded and assigned scores are based solely on the frequency of displayed behaviors; the absence of a behavior is not considered. The absence of intervention is, however, coded in the autonomy allowance scale.

Identifying codable behaviors follows a two-step process. Coders should first determine if the behavior fits the description in the scale introduction. If a behavior is determined to fit the characteristics in the scale introduction, then the coder determines the intensity of the behavior.

The score assigned for each scale is determined by the frequency and intensity of coded behaviors. Codes for all four scales are as follows:

1. The relevant behavior is not at all characteristic of the interaction. Generally, the parent either does not show any clear instances of the behavior or shows infrequent and low-intensity behavior.
2. The interaction is characterized by low-intensity behavior. Generally, the parent shows frequent low-intensity behavior. Some moderate-intensity behavior may be present, but rare.
3. Moderate-intensity behavior is somewhat characteristic of the interaction. Generally, the parent shows infrequent moderately-intense—but no highly-intense—behavior.
4. Moderate-intensity behavior is clearly characteristic of the interaction. Generally, the parent shows frequent moderately-intense behavior. Some high-intensity behavior may be present, but rare.
5. The parent shows strong behavior. The parent shows some highly-intense instances of behavior in the context of an interaction characterized by consistent moderate behavior.

A non-zero value must be given for the two scales that code parent challenging behavior. However, both overprotection and autonomy allowance code parents’ responses to children’s behavior and thus coders may assign a zero (“not applicable”) if children never engage in any eliciting behavior.

Note: This coding system is heavily influenced by Mirjana Majdandžić’s “Coding Protocol of Parenting Behavior in Parents of Toddlers” [35] described by Majdandžić et al. [29]. Construct definitions for Challenging Parenting Behavior and Overprotection are taken from her coding system, as are the differentiation between physical and expressive challenging parenting behavior and overprotection.

Challenging Behavioral Competence:

“The challenging behavioral competence (CBC) construct reflects the extent to which the parent encourages the child to go outside of their comfort zone” [35] (p. 10) and push the limits of their behavioral competence, including by taking risks. Behavioral competence refers to the ability to achieve action goals without assistance, and may be challenged when parents encourage children to add new behaviors to their repertoire or to pursue action goals through more mature means. Codable behaviors encourage children (a) to engage in behaviors beyond their current ability and/or (b) to develop cognitive abilities that directly support behavioral competence relative to ongoing tasks. Parents could challenge their children through either physical interaction (e.g., physical support during climbing) or expressions (e.g., verbal encouragement or teaching children novel solutions to problems). Both quantity and intensity of challenging behavior are considered.

CBCs that are poorly-attuned to their child’s abilities and potential and are unwelcome to the child should not be coded in this scale. Examples of poorly attuned behaviors include those that occur while the child is clearly dysregulated or which lead to dysregulation (but not necessarily lower-level frustration), those that encourage clearly dangerous behavior, or those that are clearly beyond the child’s developmental level. However, the presence of distress does not mean the CBC is inappropriate. Effective challenging behavior causes the child to go outside their comfort zone and into the zone of proximal development; this should be expected to cause some distress (but not dysregulation). Additionally, behavior that may appear intrusive to the coder may not be experienced by the child as such. For example, a child who is calmly acting toward an easy goal may welcome a parent’s prodding to attempt a more ambitious goal. Therefore, coders should use a lax criterion when deciding whether a behavior is challenging, and disregard only behaviors that are clearly poorly-attuned to the child’s current actions or beyond the child’s zone of proximal development. Because this scale is meant to complement the NICHD scales—which differentiate intrusive and sensitive behaviors—the coder should not reserve high scores on this scale for sensitive challenging parenting behavior. Additionally, purely supportive comments about behavior that don’t encourage persistence toward goals (e.g., “good job!”) do not qualify as CBC.

Provide separate numerical scores for physical and expressive challenging behavior. Physical CBC includes behaviors that involve physical contact or object-mediated physical play (e.g., tug-of-war) and that encourage children to attempt more challenging tasks than they are currently attempting. Coders should use contextual information to help determine if behaviors are intended to support the development of children’s competence or are driven by the parent’s agenda. For example, a parent who relocates a child to another area may be alerting the child to a new activity; in this context, the physical interaction is intended to present the child with a new challenge. However, a parent who relocates a child away from a potentially risky area to a safer area may be either protecting the child (in a situation with legitimate risk) or being overprotective (in a situation without legitimate risk); in this context, the behavior is not challenging.

Expressive CBC includes verbal or nonverbal expressions that encourage the child to do what they find difficult and to think in more mature ways. Coders should use contextual information to help determine if behaviors are intended to support the development of children’s competence. For example, a parent who explains a problem at a level clearly too advanced for their child may be attempting to impress an audience rather than challenging their child.

Coding:

Intensity is determined by the level of challenge, parent affect, the degree of unpredictability, the duration of activity, and the amount of physical force used. The guides below are not comprehensive. Coders should use their knowledge of typical parent behavior and use their judgment to determine intensity.

Physical CBC:

- Low intensity CBCs use physical means to provide mild challenges to children’s behavioral competence. Examples include gently and physically supporting children’s attempts toward easy ongoing action goals (e.g., holding the hand of a child who is climbing an easy incline, gently manipulating the child’s body in a task requiring physical coordination) or behaviors where the physical interaction is not clearly or effectively supporting the more challenging goal (e.g., moving the child’s hand but not explaining the goal of the intervention).
- Moderate intensity CBCs use physical means that clearly challenge children’s *demonstrated* behavioral competence but not their *potential* behavioral competence. Examples include physically supporting children to engage in an action more difficult than the ongoing action, but which the child is comfortable attempting (e.g., physically encouraging the child to climb an object they would not have climbed at that moment, manipulating the child’s body in a way that they would not have attempted naturally) or attempting to challenge the child’s potential competence but doing so ineffectively (e.g., moving the child’s hand but ineffectively explaining the goal of the intervention).
- High intensity CBCs effectively use physical means to challenge children to reach their behavioral potential. Examples include effective physical encouragement to children to accomplish a feat that they are clearly apprehensive to attempt or struggling to accomplish (e.g., succeeding at supporting a child who climbs an object despite some difficulty or resistance—but not dysregulation—on the part of the child). Coders may also consider moving moderate intensity behaviors to intense behaviors if they occur unpredictably (e.g., when the child is attending elsewhere or early in the interaction when the child may not be familiar with the space).

Expressive CBC:

- Low intensity CBCs use expressive means to provide mild challenges to children’s behavioral competence. Examples include verbally encouraging the child to persist toward an easy ongoing action goal, suggesting a more challenging task but not encouraging further efforts, encouraging children to use objects in novel ways, and using an animated facial expression or gesture to motivate the child to persist on an easy task when parental motivation seems to be required. Behaviors that may appear to be moderate intensity but which are clearly ineffective should be coded as low intensity.
- Moderate intensity CBCs use expressive means that clearly challenge children’s *demonstrated* behavioral competence but not their *potential* behavioral competence. Examples include successfully using verbal or gestural means to encourage children to engage in an action or goal more difficult than the ongoing action but which is within the child’s demonstrated abilities, asking challenging questions in the service of fostering behavioral competence, teaching the child a behavioral strategy within the child’s abilities, or attempting to challenge the child’s potential competence but doing so ineffectively (e.g., encouraging the child to reach their behavioral potential but the child disregards the comment).
- High intensity CBCs effectively use expressive means to challenge children to reach their behavioral potential. Examples include expressions that effectively push children to reach ambitious goals, scaffolding that results in creative problem-solving and/or the use of objects or activities in more sophisticated and complex ways, comments presented in an emotionally-charged tone of voice that successfully encourage the child to reach their behavioral potential, commands or forceful prodding of the child to switch tasks, teaching the child a challenging concept (i.e., the parent must persist in teaching the new concept for an extended time).

Challenging Regulatory Competence:

The challenging regulatory competence (CRC) construct reflects the extent to which the parent either creates a challenge to the child’s ongoing self-regulation or encourages the child’s regulatory efforts. Codable behaviors are those that (a) destabilize the child by creating an emotional reaction; (b) interrupt the child during an ongoing task creating an attention-regulation challenge (if the child is required to return to the task) or emotion-regulation challenge (if the child frustrated by an inability to return to the task); or (c) support or encourage the child’s regulatory efforts. High scores on this scale suggest that parent behaviors support children’s ability to regulate intense emotions or solve challenging regulatory problems. Both quantity and intensity of challenging behavior are considered.

As with challenging behavioral competence, CRCs that are poorly attuned to their child’s abilities and potential and are unwelcome to the child should not be coded in this scale. Examples of poorly attuned behaviors include those that occur while the child is clearly dysregulated or which lead to dysregulation (but not necessarily lower-level frustration), those that encourage clearly dangerous behavior, or those that are clearly beyond the child’s developmental level. Therefore, coders should use a lax criterion when deciding whether a behavior is challenging, and disregard only behaviors that are clearly poorly attuned to the child’s current actions or beyond the child’s zone of proximal development.

Coding:

Intensity is determined by the level of challenge, parent affect, the degree of unpredictability, the duration of activity, and the amount of physical force used. The guides below are not comprehensive. Coders should use their knowledge of typical parent behavior and use their judgment to determine intensity.

Low intensity CRCs are those that provide mild challenges to children’s regulatory competence or encourage children to regulate mild distress. Examples include gentle physical games (light tickling), gently eliciting new emotions through verbal or gestural means (e.g., saying “boo” in a relatively calm tone of voice), encouraging children to manage mild distress, or ineffective support for children’s attempts to manage moderate distress.

Moderate intensity CRCs are those that clearly challenge children’s regulatory competence, introduce some risk where mild distress may be justified, or encourage children to regulate obvious distress. Examples include brief physical games that require the child to use some amount of force (e.g., tug-of-war, chasing) or feel momentary distress (e.g., gentle tossing in the air), longer bouts of gentle physical play, more intense attempts at destabilization that either do not elicit a strong reaction or do not interrupt intense focus, gentle teasing (e.g., playfully saying “can you really do that?” while the child is engaged in a mild struggle), effective support for children’s attempts to manage moderate distress, or ineffective support for children’s attempts to manage extreme distress.

High intensity CRCs are those that push children to the limit of their regulatory competence or are effective in encouraging children to regulate intense emotions. Examples include extended physical games that involve the use of force and a change in the child’s emotional state (e.g., tickling that leads to intense laughter, chasing that involves running, wrestling), destabilization that interrupts a child who is engrossed in a task and/or results in a strong reaction but *not* dysregulation, teasing the child in ways that more forcefully challenge the child’s competencies (e.g., saying “no way, you can’t climb all the way up there” or “I don’t think you can solve such a difficult puzzle all by yourself” where the intent is clearly to spur the child to reach a more advanced goal, but not belittle the child).

Overprotection:

“Overprotection reflects the extent to which the parent conveys exaggerated worry or concern for the child’s wellbeing and safety. During coding, attention is paid to how carefully the parent handles the child and to what extent the parent shows behavior aimed at protecting the child” [35] (p. 12). Note that behavior that is protective of children’s safety during times of legitimate potential for harm is not considered overprotective. Both quantity and intensity of overprotection are considered.

Coding:

Provide separate numerical scores for physical and expressive overprotection.

Examples of behaviors that indicate physical overprotection are those that use physical force to restrict child movement. Low, moderate, and intense ratings are given based on the level of protection inherent in the parent behavior, the degree of legitimate risk, duration of activity, parent affect, and child affect.

- Low intensity examples include briefly restraining the child when the risk of danger is small, redirecting movement away from perceived danger despite small degree of risk (and with no resistance from the child), or maintaining constant close physical proximity to the child and willingness to intervene during periods of no risk of danger.
- Moderate intensity examples include restraining the child despite no clear sign of risk, restraint or redirection from low-risk situations which results in some child resistance, or hovering over the child in a pose that suggests readiness to intervene during periods of minimal risk to the child.
- High intensity examples include firmly holding the child while they attempt to pull free and attempt an activity with no clear sign of risk, and picking up the child in order to either redirect movement or remove them from the situation.

Examples of behaviors that indicate expressive overprotection are those that use verbal or facial expressions to restrict child movement.

- Low intensity examples include calm expressions of concern (e.g., reminders to be cautious, “hold on,” mild facial expressions of apprehension), or warnings against proceeding with an activity (e.g., “I don’t think you should do that”), or disapproving facial expressions when children are engaging in a task) when the risk of danger is small.
- Moderate intensity examples include expressions of concern or warnings against proceeding with an activity, either when those activities show no clear sign of risk or when those expressions are given with a worried tone of voice.
- High intensity examples include: expressions (e.g., gasping, very fearful expressions, “watch out!”) with emotional displays that signal a risk of impending danger that substantially exaggerates the degree of risk, explicit prohibitions (“stop!”) against proceeding with a safe activity, or explicit statements (“that’s scary,” “that makes me nervous”) about the parent’s concern for the child’s safety in safe activities.

Note: When assigning an overprotection score, parents whose children never attempt risky activities (for reasons of their own choosing, not because of parental overprotection) can be given a zero.

Autonomy Allowance:

Autonomy allowance describes behavior that allows children to autonomously pursue activities that are outside of their comfort zone, beyond their current abilities, or contravene typical expectations of behavior, by simply attending to the child’s activities while adopting a stance of non-intervention. Parents who allow children to work autonomously due to being detached and unaware of the child’s activities are not considered to be demonstrating autonomy allowance; there must be evidence that parents are visually or aurally attending to the child’s activities to determine that non-intervention is the result of a parent decision to allow autonomy. Autonomy allowance also occurs when parents allow children to act in unconventional—but not inappropriate—ways without correcting the behavior. Intervention refers to parent behaviors that insert their own agency into the process of task completion (i.e., the parent completes steps that the child is capable of completing or gives instructions that the child would know).

Low, moderate, and intense ratings are given based on the degree of the child’s struggle to make progress, the parent’s intervention latency, the extent to which the behavior contravenes typical expectations that parents have of children’s behavior, and the type of intervention. At low levels, the parent initially does not intervene, but may intervene quickly after the child does not make progress. At high levels, the parent maintains attentive non-interference for extended periods despite the child’s continued lack of progress, signs of struggle, or signs of distress. The coder should take intervention latency into account; parents who attend to the child’s struggle for a considerable amount of time before reaffirming the child’s skill may still get scores reflecting high levels of autonomy allowance.

Coding:

- Low intensity examples include situations in which the parent allows the child to work on easy tasks without intervention only until the child shows signs of struggle, after which intervention is swift; any situation in which parents engage in unnecessary physical intervention after allowing independent work (i.e., a lengthy period of autonomy allowance ended by unnecessary physical intervention cannot receive an intensity rating above low); or maintaining proximity to the child during low-risk activities but not indicating a desire to intervene.
- Moderate intensity examples include situations in which the parent allows the child to work on easy tasks with no intervention for long periods of time and/or waits briefly before intervening when the child shows signs of struggling on a task; refraining from unnecessary physical intervention—but still offering verbal interventions—during challenging behavioral tasks; comments about the child’s lack of need for parent assistance during tasks within the child’s demonstrated competence, allowing the child to disregard parent suggestions or directives, or maintaining close proximity—but not hovering in manner suggesting a desire to intervene—during physically challenging tasks.
- High intensity examples include situations in which the parent allows the child to work on challenging tasks with no intervention or minimal intervention for long periods of time; waiting until signs of significant distress (but not dysregulation) before even verbal intervention; comments about the child’s lack of need for parent assistance on tasks that challenge the child’s potential competence, or keeping physical distance even during significant physical challenge. These parents are content to let their child encounter any struggle autonomously as long as the parent believes that goal-completion is within the child’s ability.

Notes: (1) parents who intervene when children show signs of dysregulation should not be penalized on their score; (2) when assigning an autonomy allowance score, parents whose children never attempt activities outside of their comfort zone or beyond their current abilities can be given a zero.
